# GAS6 Receptor Status Is Associated with Dormancy and Bone Metastatic Tumor Formation

**DOI:** 10.1371/journal.pone.0061873

**Published:** 2013-04-24

**Authors:** Russell S. Taichman, Lalit R. Patel, Rachel Bedenis, Jingcheng Wang, Savannah Weidner, Taibriana Schumann, Kenji Yumoto, Janice E. Berry, Yusuke Shiozawa, Kenneth J. Pienta

**Affiliations:** 1 Department of Periodontics and Oral Medicine, University of Michigan School of Dentistry, Ann Arbor, Michigan, United States of America; 2 Departments of Internal Medicine and Urology, University of Michigan School of Medicine, Ann Arbor, Michigan, United States of America; 3 Departments of Urology, Oncology, Pharmacology and Molecular Sciences, Brady Urological Institute, Baltimore, Maryland, United States of America; The University of Texas M.D Anderson Cancer Center, United States of America

## Abstract

Disseminated tumor cells (**DTCs**) are believed to lie dormant in the marrow before they can be activated to form metastases. How DTCs become dormant in the marrow and how dormant DTCs escape dormancy remains unclear. Recent work has shown that prostate cancer (PCa) cell lines express the growth-arrest specific 6 (GAS6) receptors Axl, Tyro3, and Mer, and become growth arrested in response to GAS6. We therefore hypothesized that GAS6 signaling regulates the proliferative activity of DTCs in the marrow. To explore this possibility, *in vivo* studies were performed where it was observed that when Tyro3 expression levels exceed Axl expression, the PCa cells exhibit rapid growth. When when Axl levels predominate, PCa cells remain largely quiescent. These findings suggest that a balance between the expression of Axl and Tyro3 is associated with a molecular switch between a dormant and a proliferative phenotype in PCa metastases.

## Introduction

Prostate cancer (**PCa**) cells have an astonishing ability to disseminate to the bone marrow. Once there, disseminated tumor cells (**DTCs**) may lie dormant for years, undetected by standard clinical methods. How DTCs become dormant in the marrow, and how proliferation is activated to produce skeletal metastases remains unknown [1].

Hematopoietic stem cells (**HSCs**) are multipotent stem cells that give rise to all mature blood cell types. HSCs normally reside in the bone marrow of adult mammals where their fate is tightly balanced between proliferation and quiescence [2]–[5]. HSC quiescence is regulated by microenvironmental controls derived from cellular ‘niches’ comprised predominately of osteoblasts, endothelial cells and other marrow elements [6]. One molecule that regulates HSC quiescence is growth arrest specific 6 (**GAS6**)[7] which in marrow is secreted by osteoblasts [8]. GAS6 binds to the tyrosine kinase receptors Axl (from the Greek word ‘anexelekto,’ or uncontrolled, also named ARK, Tyro7, Ufo) [8], [9], Tyro3 (Sky, Rse, Brt, Dtk, Tif, Etk2, Rek) and Mer (MerTK, c-Eyk, Nyk, Tyro12) [10]. In many systems, GAS6 inhibits cellular proliferation, promotes survival and increases resistance to chemotherapy [11]. In other systems, GAS6 stimulates proliferation [12], [13]. For HSCs, it appears that a balance between the expression of Axl, Tyro3 and Mer determines whether the cells remain quiescent or proliferate [14]–[16].

Recently we reported that, PCa cells target and engage the HSC niche during dissemination [17]. We also reported that when PCa cells bind to osteoblasts in the niche they increase their expression of Axl and GAS6 signaling inhibits PCa proliferation [8]. These findings suggest that once DTCs enter the niche, interactions between GAS6 and its receptors may regulate PCa dormancy. Therefore, this work attempted to correlate the expression of GAS6 receptors with a quiescent state in PCa cells recovered from primary tumor sites versus DTCs isolated from the marrow. The data suggest that when PCa expression of Axl is higher than Tyro3, DTCs in the marrow are found to be predominantly quiescent. Conversely, when Tyro3 expression levels exceed Axl expression, proliferation is the predominant behavior.

## Methods

### Cell Culture

Human PCa cell lines (PC3, DU145, LNCaP), primary human fetal osteoblasts (hFOB) and MG-63 human osteosarcoma cell lines were obtained from the American Type Culture Collection (Rockville, MD). Luciferase expressing PC3 and DU145 cell lines were established by lentiviral transduction [18]. Human bone marrow endothelial cells (HBME) were established as previously reported [19]. PCa cell lines and marrow cell lines were cultured in RPMI 1640 (Invitrogen, Carlsbad, CA) and Dulbecco’s minimum essential medium (DMEM, Invitrogen), respectively. All cultures were supplemented with 10% fetal bovine serum (FBS; Invitrogen), 1% penicillin-streptomycin and 1% L-glutamine (Invitrogen). The cultures were maintained at 37°C, 5% CO2, and 100% humidity.

### Fluorescence Activated Cell Sorting (FACS)

Cell sorting and flow cytometry analyses were performed using the University of Michigan Flow Core’s BD Vantage, BD Aria II, or the Beckman Coulter Cyan. Unconjugated-Axl, unconjugated-Tyro3, PE-Tyro3 and PE-Mer (R&D system, Minneapolis, MN), FITC-HLA-ABC (Biolegend, San Diego, CA), unconjugated-Ki67 (Abnova, Walnut, CA.) and isotype controls (Biolegend, R&D, Abnova) were used in our studies. The unconjugated antibodies were custom conjugated using Lightning Link antibody conjugation kits (Novus Biologicals, Littleton, CO) per manufacturer instructions for the fluorophores APC-Cy7, PE-Cy5, and Atto390. After conjugation, all antibodies were diluted to a working concentration of 20 µg/mL in DPBS. Isotype controls (Biolegend, R&D, Abnova) were similarly conjugated.

Murine bone marrow was flushed from the femurs, tibias, and humeri with DMEM. Cells were washed and resuspended as single cell suspensions in MACS buffer supplemented with BSA (Miltenyi Biotec). Single cell preparations were incubated first with a Lineage Cell Depletion Kit magnetic labeling system, with biotinylated anti-Lineage (CD5, CD45R (B220), CD11b, Gr-1 (Ly-6G/C), and Ter-119) antibody cocktail and anti-Biotin MicroBeads (Miltenyi Biotec, Auburn, CA), and then depleted of hematopoietic lineage cells using MS and LS columns (Miltenyi Biotec) per manufacturer instructions. Staining for flow cytometry was subsequently performed on lineage depleted fractions enriched for PCa.

### Western Blots

Lysates recovered from HEK293 cells overexpressing human Tyro 3 or vector control were purchased from Origene USA (Rockville, MD). PCa cells were prepared in lysis buffer (CelLytic MT Mammalian Tissue Lysis Reagent, Sigma-Aldrich) and the protein concentration was quantified by DC Protein Assay Kit (Bio-RAD). The lysate of TYRO3-overexpressing HEK293 cells (5 µg of protein per lane) was used as a positive control. Cell extracts (30 µg of protein per lane) were loaded and separated on SDS-PAGE (4–20% Bis-Tris gradient gels, Invitrogen) and transferred to a PVDF membrane. The membranes were incubated with 5% milk for 1 hr and incubated with anti-Human TYRO3 antibody overnight at 4°C. Primary antibody was used at a 1∶500 ratio with 5% dry milk. Blots were incubated with peroxidase-coupled secondary antibodies (Promega, Madison, WI) for 1h at a ratio of 1∶3000. Protein expression was detected with SuperSignal West Pico Chemiluminescent Substrate (Thermo Scientific, Rockford, IL).

### Proliferation Assays

Proliferation was assessed using the ACEA RT-CES system monitoring the electrical impedance of cells in each well of the apparatus as a real-time measurement of cell quantity. Cells were serum starved overnight, resuspended in media supplemented with 0.5% FBS and GAS6, and plated at a density of 5000 cells/well. Test doses of GAS6 were diluted in vehicle ranging from 0–4 µg/ml final doses. Electrical impedances were measured in each well every 15 minutes for 120 hours and reported in ACEA Index units.

### Implantation of Scaffolds Containing PCa Cell Lines

Adult male SCID mice (5- to 7-week old CB.17. SCID; Taconic, Germantown, New York) were used as tumor recipients and were housed under constant humidity and temperature, with 12-hour light, 12-hour dark cycles and monitored daily. Each group consisted of a range of 8–12 animals. The animals were anesthetized by isofluorane inhalation. Human PCa cells were implanted in sterile collagen scaffolds (3×3×3 mm, Gelfoam®, Pharmacia & Upjohn Co. New York, NY). Immediately prior to implantation, 2.5×10^5^ luciferase labeled PCa cells were resuspended in 20 µl of RPMI 1640 containing 5% FBS and allowed to adsorb into the scaffolds. Single mid-dorsal incisions were made for subcutaneous (*s.c.*) implantation of the tumor-bearing scaffolds. Each incision was closed with surgical clips. The animals were monitored by bioluminescent imaging (BLI) weekly until tumors were observed in >80% of implanted mice [20]. Thereafter, the *s.c.* tumors were resected and the animals were reimaged. All mice growing tumor at the primary site after resection were excluded from the study. Cohorts of mice were followed for 5 months post tumor removal. Upon achieving the endpoints of the study, the animals were anesthetized and phlebotomy performed by intracardiac puncture. All procedures were approved by the University of Michigan Committee on the Use and Care of Animals (UCUCA).

### Intracardiac Injections

Intracardiac injections (i.c.) of luciferase-labeled PCa cells were performed in 6–8 week-old male CB.17. SCID mice under 3% isofluorane anesthesia (Abbott Laboratories, North Chicago, IL). Left ventricular cardiac injections were performed with 2×10^5^ cells suspended in 100 µl of PBS using a 27-gauge needle.

### Immunohistochemistry

To detect human cells grown in mice, anti-human HLA-A,B,C antibody (BioLegend, San Diego, CA 92121) was conjugated using the Zenon Alexa Fluor728 mouse IgG_2a_ labeling kit. To detect human Axl protein, purified Goat IgG antibody (R & D Systems, Minneapolis, MN 55413) was conjugated using the Zenon Alexa Fluor 555 Goat IgG labeling kit (Invitrogen, San Diego, CA 92121). For detection of Tyro3, the antibody (Abcam Cambridge MA 02139) was conjugated using the Zenon Alexa Fluor 555 Rabbit IgG labeling kit (Invitrogen, San Diego, CA 92121). 7 µm thick paraffin sections were generated per tissue, and antigen retrieval performed with a pepsin solution at 37°C for 15 min. followed by washing with PBT (PBS plus 0.2% Triton X-100) for 5 min at room temperature. Each section was blocked with Image-iT FX signal enhancer (Invitrogen, San Diego, CA 92121) for 30 minutes before fluorescence-labeled primary antibodies were applied at room temperature for 2 hours in the dark. Subsequently, the sections were washed twice by submersion in PBS for 10 min., subjected to post-stain fixation with 10% formalin (Sigma), and mounted with ProLong Gold anti-fade reagent with DAPI (Invitrogen, San Diego, CA 92121). Images were taken with Olympus FV-500 confocal microscope.

### Statistical Analysis

Numerical data are expressed as means ± standard deviation. Statistical differences between the means for the different groups were evaluated with Instat 4.0 (GraphPAD software) using one-way analysis of variance (ANOVA) with the level of significance at *p*<0.05.

## Results

### Expression of GAS6 Receptors by PCa Cells

**Figure 1:**
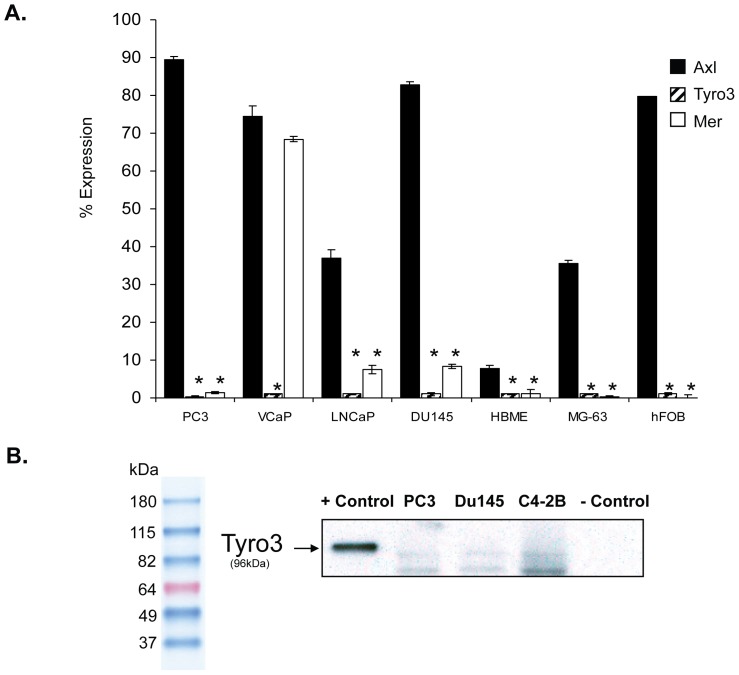
GAS6 receptor expression by PCa cell lines. (A) FACS analysis for GAS6 receptors. The human PC3, VCaP, LNCaP, and DU145 PCa cell lines, human bone marrow endothelial cells (HBME), MG-63 human osteosarcoma cell line and primary human fetal osetoblasts (hFOB) were stained with antibodies targeting Axl, Tyro3 or Mer and the % expression were compared to IgG controls for each antibody for n = 3 samples. The data is expressed as avg. ± s.d. *p<0.05 compared to expression of Axl for each cell type. (B). Western blot analysis for Tyro3. Since Tyro3 expression was not detected in (A) by FACS, validation by Western Blot of PCa cell lysates (30 µg) was performed. Lysates (1 µg) recovered from HEK293 cells which over expressed of human Tyro 3 (or vector control) were used as positive and negative controls for Tyro3 expression. Normalization of PCa cell extracts was evaluated by staining with antibody to ß-Actin (not shown).

Previously we showed that binding of PCa to annexin alters the expression of GAS6 receptors on PCa cell lines ([8]). Here, we explored which of the GAS6 receptors are expressed by PCa cell lines under basal culture conditions. For these studies, cells which produce predominantly osteolytic (PC3 and DU145) [21] and mixed osteoblastic (LNCaP [22], [23] and VCaP [24] bone phenotypes were evaluated for their expression of Axl, Tyro3, and Mer by FACS. Human bone marrow endothelial, osteosarcoma (MG-63), and fetal human osteoblast (hFOB) cells were included as controls. All of the human PCa cell lines expressed high levels of Axl when directly recovered from culture (**Figure 1A**). VCaP, LNCaP, PC3 and DU145 expressed detectible but low levels of Mer (**Figure 1A**). None of the PCa cell lines expressed significant levels of Tyro3 (**Figure 1A**), which was confirmed by Western blot (**Figure 1B**).

### Axl and Tyro3 Expression during Tumor Dissemination and Following the Induction of Dormancy

**Figure 2:**
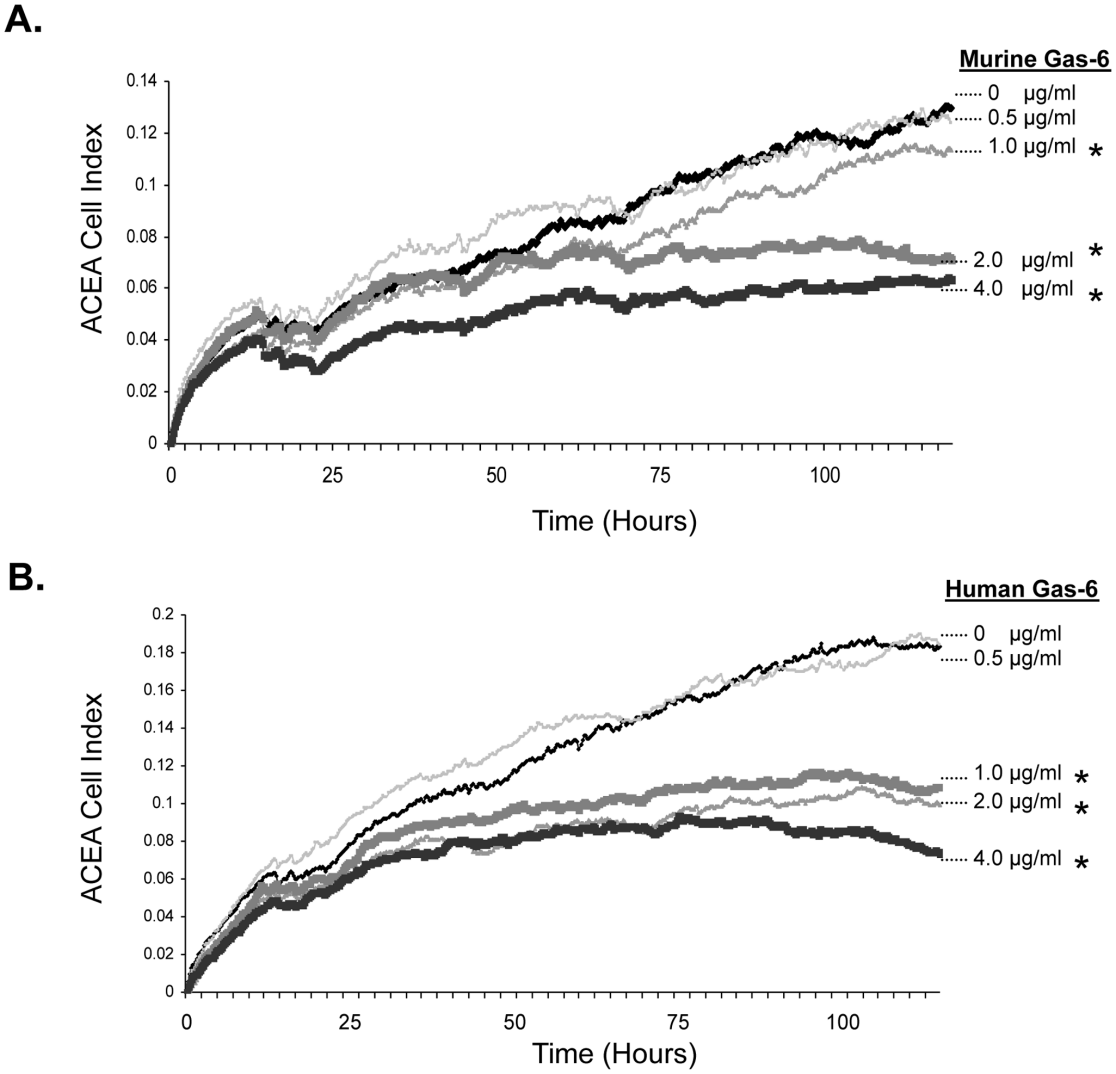
Murine and human GAS6 inhibits growth of human PC3 cells in vitro. Human PC3 cells were plated at 2,500 cells/well into 96-well flat bottom tissue culture with increasing concentrations of (A) murine or (B) human GAS6 (0, 0.5, 1.0, 2.0, and 4.0 µg/ml). Proliferation over time was quantified by impedance measurements in the ACEA RT-CES real-time cellular proliferation assay system. n = 6 wells. The data is expressed as avg. ± s.d. over time. *p<0.05 compared to 0 µg/ml GAS6.

Previously we indicated that lower levels of GAS6 expression correlated with PCa proliferation in osseous sites ([8]). Prior to *in vivo* murine studies, we examined whether murine GAS6 had activity on human cells in culture by treating PC3 cells with murine and human GAS6. As demonstrated in **Figure 2A and B**, the PC3 numbers were reduced as the concentration of both murine and human GAS6 increased. This suggests that murine GAS6 can regulate proliferation of human PCa cell lines.

**Figure 3:**
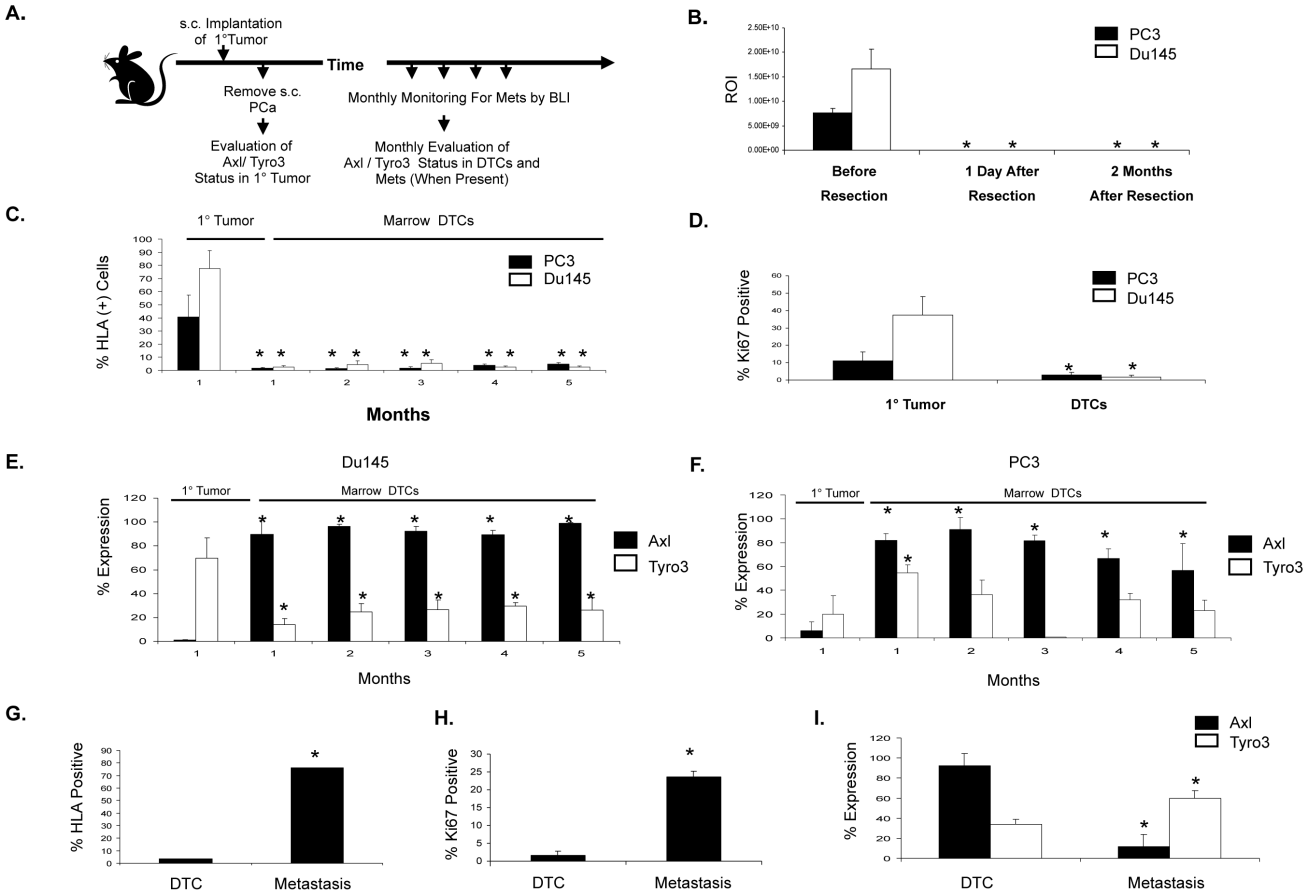
Axl and Tyro3 expression during experimental PCa progression. (**A**) Experimental model. Human PCa cell lines (PC3^Luc^, DU145^Luc^) were implanted *s.c.* into male SCID mice as a model of a primary (1°) tumor development, and removed after 1 month. At monthly intervals thereafter human PCa cells were identified by anti-HLA staining; proliferative status (Ki67 staining) and Axl or Tyro3 levels were evaluated by FACS. (**B**) When *s.c.* tumors were identified and removed, and the animals were reimaged 1 day later and at 2 months by BLI to determine if regrowth had occurred at the primary tumor bed. ROI = Region of interest (**C**) Percent expression of HLA by lineage depleted (Lin^-^) marrow cells or by primary tumor cells at 1 month. (**D**) Percent expression of Ki67 by lineage depleted (Lin^-^) marrow cells or by primary tumor cells at 1 month. Percent expression of Axl or Tyro3 by primary tumor cells established with (**E**) DU145 or (**F**) PC3 cells or by DTCs recovered from marrow over time. (**G**) At 5 months bone metastatic lesions were detected in animals initially implanted with *s.c.* DU145. Percent expression of HLA by DTCs isolated from the non-metastatic limb or in the metastatic lesion. (**H**) Expression of Ki67 by HLA expressing DTCs, or by PCa cells recovered from bone metastatic lesions. (**I**) Percent expression of Axl and Tyro3 by HLA expressing DTCs isolated from the non-metastatic limb or by cells recovered from the metastatic lesion. *p<0.05 compared to expression of each receptor in the primary tumors.

Recently we developed an *in vivo s.c.* model to track early dissemination events from solid tumors [18]. Human PCa cell lines implanted into immune deficient mice can be detected and recovered from different organs using antibodies to human HLA antigens. Importantly, if the primary tumors are removed, some of the animals develop metastatic bone lesions over the course of several months (18]. We therefore used this model to evaluate the expression of Axl and Tyro3 in both the primary *s.c.* tumors and DTCs in boney sites over time (**Figure 3A**) [25], [26], after first ensuring that recurrence in the primary site had not occurred (**Figure 3B**).

Animals were sacrificed over a 5-month period and bone marrow DTC numbers were quantified by FACS. Overall, less than 5% of the total marrow cells expressed human antigens as determined by anti-HLA antibodies (**Figure 3C**), and of those, fewer than 5% expressed the proliferation marker Ki67 (**Figure 3D**). In contrast, cells recovered from the primary tumor site expressed significantly higher levels of Ki67 (**Figure 3D**). These data suggest that DTCs in the marrow proliferate less than cells isolated from the primary tumors. 70% of the DU145 primary tumor cells expressed Tyro3, while Axl expression was considerably less than observed in cell culture (**Figure 3E** and **Figure 1**). In the marrow, the percent of DU145 DTCs expressing Tyro3 was significantly reduced compared with cells isolated from the primary tumors. In contrast, Axl expression was significantly increased by DTCs compared with cells from the primary tumor, and remained elevated by ∼80% of the DTCs recovered over five months (**Figure 3E**). In the PC3 system, Axl expression was significantly decreased in the primary tumor compared to cells grown *in vitro,* while Tyro3 levels were elevated (**Figure 3F** and **Figure 1**). DTCs recovered during the entire animal study expressed elevated levels of Axl compared with cells from the primary tumors. In addition, Tyro3 expression by PC3 cells followed the same basic pattern observed in the DU145 system. Although expression was more variable in PC3 when compared to DU145, Tyro3 levels were still higher than Axl levels in the primary tumors (**Figure 3F**). Likewise, PC3 DTCs expressed higher levels of Axl than Tyro3, although the expression levels of both receptors were higher than the s.c. primary tumor levels (**Figure 3F**).

Fortunately, PCa recurrence is a rare event in most men, even when DTCs are present in the marrow [25], [26]. Likewise, in the *s.c.* model, when metastases do occur (less than 10% of the mice) they typically are identified months after inoculation [18]. Two animals implanted with DU145 cells developed defined metastatic identified by BLI at 4 months. We therefore compared the percentage of HLA expressing cells in the femur with metastatic disease to DTCs isolated from the femur without disease (**Figure 3G**). Here one of the metastases was processed for immunohistochemistry and the other for FACS analysis. When tumor was present in the femur, more than 70% of recovered lineage depleted bone marrow cells were of human origin compared to less than 3% of the control femur as examined by FACS. Nearly 23% of the HLA^+^ cells isolated from the tumor-bearing femur expressed Ki67 (**Figure 3H**). Fewer than 3% of the DTCs recovered from the control limb expressed Ki67 (**Figure 3H**). Importantly, 92% of the DTCs expressed Axl verses 12% of the cells recovered from the metastatic lesion (**Figure 3I**). Tyro3 levels on the other hand increased from 34% to 60% during the progression from DTC to metastasis (**Figure 3I**).

**Figure 4:**
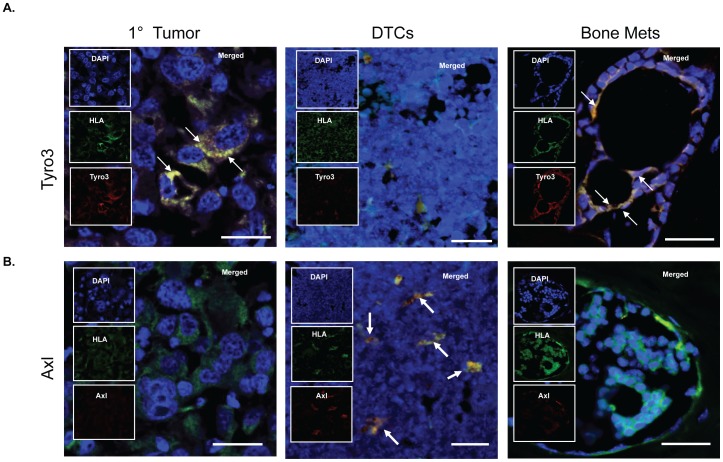
Expression of Axl and Tyro3 during experimental PCa progression by PC3 cells. Immunohistochemistry for Axl and Tyro3 expression during PC3 progression to metastasis from a *s.c.* primary tumor. (**A**) Primary tumor, marrow DTCs and femur metastasis stained with DAPI nuclear stain (blue), human HLA antigens (green), and Tyro3 (red). Double stained cells for HLA and Tyro3 appear yellow or white when merged. Inserts represent each individual stain merged in the larger panel. (**B**) Sections stained with DAPI nuclear stain (blue), human HLA antigens (green), and Axl (red). Double stained cells for HLA and Axl appear yellow or white when merged. Bar = 100 microns.

Similar to the results seen by FACS, immunohistochemistry for Tyro3 expression showed significant co-localization with human HLA expression in the primary *s.c.* tumors and when bone metastasis developed (**Figure 4A**). Conversely, Tyro3 expression by DTCs was significantly down regulated (**Figure 4A**). On the other hand, Axl expression was elevated in DTCs but its expression was down regulated in human PCa cells at the primary tumor sites or in metastatic bone lesions (**Figure 4B**).

### Expression of Axl and Tyro3 in Disseminated Metastatic Disease

**Figure 5:**
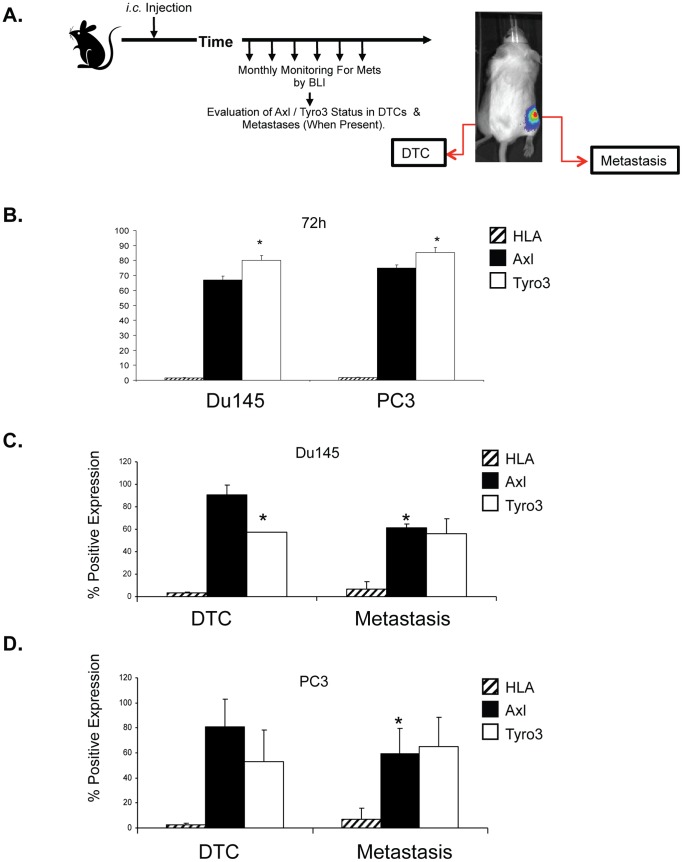
Expression of Axl and Tyro3 following intracardiac injection and establishment of disseminated disease. (**A**) Experimental model. Human PCa cell lines (PC3^Luc^ and DU145^Luc^) were injected *i.c.* into SCID mice and PCa cells were recovered at (**B**) at 72h. * *p*<0.05 compared to the expression of Tyro3 to Axl. As bone metastatic lesions were identified by BLI, paired limbs were flushed and human PCa cells were identified by anti-HLA staining. Axl or Tyro3 levels were evaluated by FACS for (**C**) Du145 and (**D**) PC3 cells. *p<0.05 compared to the expression of each receptor by paired DTC sample.

The major advantage of the *s.c.* tumor metastasis model is that disseminated disease can be modeled and studied from the perspective of dormancy. Like men with DTCs in their marrow, few mice developed overt metastatic bone lesions over the course of our multi-month study. Injections of tumor cells directly into the left cardiac ventricle affords a model of tumor metastasis that results in the development of skeletal metastases on a more frequent basis ([27]). Therefore we turned to the intracardiac injection model to further explore Axl and Tyro3 levels under conditions where bone lesions are more likely to occur (**Figure 5A**).

After i.c. injections, the animals were sacrificed at 72h or when skeletal metastases developed. At 72h, the number of DTCs detected by HLA staining was equivalent for both Du145 and PC3 injected animals. At 72h, both PC3 and Du145 DTCs expressed significantly Tyro3 than Axl (**Figure 5B**). Twenty percent of the Du145 cell injected animals developed skeletal metastases at 6 weeks. At 10 weeks, 10% of the PC3 injected animals developed tibia/femur bone lesions. At these time points, respectively, animals which had developed skeletal lesions were sacrificed and the DTCs were recovered from the tumor affected and non-affected legs for comparison. Nearly 80% of the DTCs recovered from the marrows of the non-affected limbs expressed Axl (**Figure 5C, D**). In those limbs where metastatic bone lesions developed, a significant reduction in the expression of Axl was observed compared with DTCs isolated from the non-affected limb occurred (**Figure 5C, D**). Tyro3 levels, however remained unchanged (**Figure 5C, D**).

## Discussion

Many men are deemed “cured” of organ confined PCa when treated with surgery, radiation and/or chemotherapy. Yet for those who suffer a relapse, the bone marrow is often the primary site of recurrence, which can happen years, and in some cases decades after the initial curative treatment ([28]). Recurrence in the bone marrow is only possible if some of the PCa cells from the primary tumor disseminated out of the prostate and “homed’ to the bone marrow [25], [26], [29], [30]. Recent work by our group and others has helped to define some of the mechanisms used by PCa DTCs to target and take hold in the bone marrow [31]. Considerable knowledge has also been gained as to how PCa cells proliferate in response autocrine and paracrine factors derived from the bone marrow microenvironment [32]. Yet once in the marrow, very little is known about what happens to tumor cells over the months, years, and decades when DTCs lie dormant.

We have focused on the ability of GAS6 to regulate PCa growth in the marrow. Our previous studies have demonstrated that when PCa cells bind to niche osteoblasts, they increase their expression of the GAS6 receptor Axl, and that GAS6 inhibits PCa cell growth and confers resistance to chemotherapy *in vitro* [8]. Moreover, when GAS6 is specifically removed from the bone marrow microenvironment, PCa cells grow more robustly [8]. Likewise, the prevalence of experimental metastases in skeletal sites inversely correlates with GAS6 levels [33]. These findings suggest that once DTCs enter the marrow, GAS6 signaling may regulate the proliferative activity of PCa cells.

**Figure 6:**
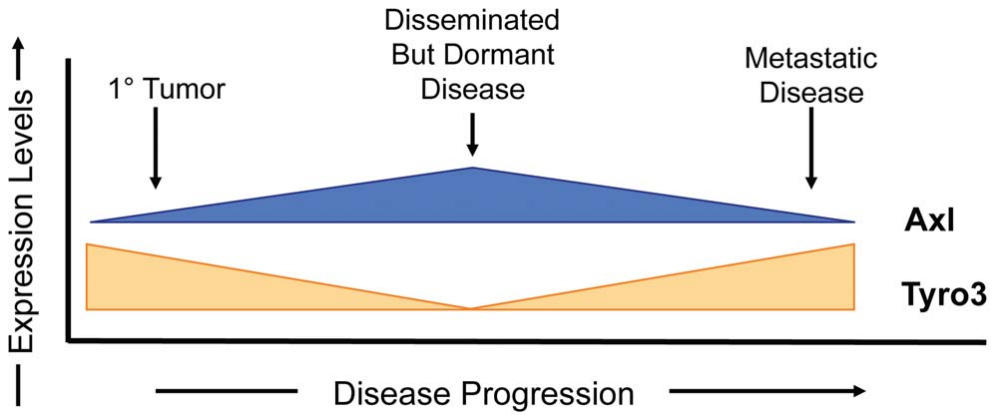
Conceptual model of Axl and Tyro3 expression during PCa progression to metastasis. Conceptual model demonstrating for Axl and Tyro3 expression during prostate cancer progression to metastasis. When Axl expression by PCa cells predominates, GAS6 inhibits growth. When Tyro3 expression predominates or when Tyro3 and Axl expression is equivalent, proliferation appears to be the predominant response of PCa to GAS6.

The major findings of this study are that Axl and Tyro3 expression are correlated with tumor growth in animal models of metastatic PCa disease (Model: **Figure 6**). Specifically, we found that Axl was down regulated by tumor cells implanted into *s.c.* spaces in a model of primary tumor growth, compared to the expression *in vitro*. PCa cells which are shed from a primary *s.c.* space into the marrow increased their expression of Axl. In contrast, little if any Tyro3 was expressed *in vitro* by most tumor cell lines. Yet, when these same cells were placed under the skin of SCID mice, Tyro3 levels increased significantly. When the Tyro3^Hi^, Axl^Low^ cells arrived in the marrow, however, Tyro3 expression was down regulated and Ki67 staining decreased suggesting that proliferation was reduced. Later, the cells shifted back to a Tyro3^Hi^ Axl^low^ phenotype in the rare instances that metastatic bone lesions were identified. To increase the chances of observing changes in the Axl/Tyro3 phenotype of DTCs as they progress towards metastasis, we turned to the *i.c.* model of dissemination. Here, DTCs predominantly express Axl, while Tyro3 remained relatively high compared to DTCs recovered from the *s.c.* model. However, when these same cells develop into tumors, Axl expression decreased while Tyro3 expression remained unchanged.

Many models of metastasis sidestep several of the early metastatic events required for dissemination by placing tumor cells directly into the circulation [18]. Therefore one of the major advantages of our *s.c.* micrometastasis model is that it mimics many of the steps used by tumors to generate DTCs and disseminated disease [18]. This advantage is coupled with the limitation of low frequencies of metastatic bone lesion development. After careful consideration we have, however, concluded that the *s.c.* model is a fair representation of the majority of men diagnosed with PCa, as most marrow DTCs remain dormant and do not develop into metastases. This conclusion is based on the findings that up to 30% of males over the age of 50, and as many as 80% of males over 80, have undiagnosed PCa [34]. However only ∼ 3% of men die from PCa [35], but when they do, bone metastases are the major cause of mortality [28]. It is therefore critical to note that as 72% of patients diagnosed with PCa, regardless of pathologic grade, have DTCs in their marrows, clearly most of these DTCs must remain dormant and few if any progress to metastatic lesions [26]. In order for an experimental model to be a valid representation of clinical experience, few skeletal metastases should develop. Therefore, although low metastatic lesion frequency may at first appear to be a limitation, it is likely that this may prove to be a highly representative, and arguably an accurate model of the disease.

Recently we published that Axl expression in tissue microarrays (TMA) increased during tumor progression[8]. Unfortunately, with TMAs originated from overt lesions, the data sets by nature do not address what happens on an individual cell level when PCa cells lie dormant as DTCs in the marrow. To answer these questions, DTCs from men will need to be isolated and similarly interrogated. The question remains as to which receptors GAS6 targets to regulate growth in the marrow. As a result, we sought to explore the hypothesis that the balance in expression of the GAS6 receptors Axl and Tyro3 regulates tumor cell proliferation and dormancy.

At present, it is not known what regulates the expression of either Axl or Tyro3. In recent work we have demonstrated that knockdown of Axl expression results in a decrease in the expression of the transcription factors snail, slug and N-cadherin, representing several mesenchymal markers. At the same time, expression knockdown of Axl enhances the expression of the epithelial marker E-cadherin in PC3 and DU145 cells [36]. These findings suggest that Axl may participate in an epithelial to mesenchymal transition linked to metastasis. We also noticed that stimulation with GAS6 down-regulated Axl expression which is not seen when the cells are in hypoxic conditions [36]. This is interesting, given that under basal conditions the normal physiologic oxygen tension in marrow is thought to be hypoxic. Particularly at the level of the endosteal/osteoblastic niche, suggesting that a low O_2_ environment provides a survival advantage for HSCs to maintain their stemness [37]. Moreover, immunochemical staining of human PCa tissue microarrays showed that GAS6 and Hif1-α (a known indicator of hypoxia) are co-expressed at levels that are elevated in PCa and in bone metastases compared to normal tissues^26^. Given the (i) association of Axl with dormancy in this study, (ii) the observation that Axl expression may become stabilized in hypoxic environments [36], (iii) the production of GAS6 by osteoblasts [8] and, (iv) the hypoxic nature of the endosteal niche[38], we hypothesize that dormancy is in part regulated by this unique niche in a manner akin to HSC-quiescence. Certainly further inquiry into the mechanistic underpinnings behind microenvironmental HSC-quiescence and DTC-dormancy in the marrow is warranted.

This study also raises additional questions pertaining to reports that suggest GAS6 stimulates[39] or inhibits proliferation of PCa cells [8]. In fact, recent findings demonstrate that blockage of Axl inhibits proliferation, invasion and migration in PCa cell lines suggesting that Axl expression is linked with proliferation[40]. From our studies it remains unclear how GAS6 regulates *in vitro* growth by cells predominantly expressing Axl, while the same PCa cells when recovered from animals with low proliferative scores predominantly express Axl. Perhaps it is instructive to consider the role of Axl in other systems where it is a regulator of endothelial cell migration, proliferation, and tube formation [13]. Axl is also critical for the ability of breast cancer cells to form tumors *in vivo* [13]. Similar results have been shown in glioblastoma and pancreatic cancers where Axl expression is elevated [41]–[45]. Yet Axl signaling is also known to increase survival of melanoma cells by limiting mitosis and increasing survival [46]. These observations underscore the inherent difficulties in studying the GAS6 system and suggest that either receptor cross-talk and/or additional signaling molecules are required.

Several molecules are likely to play a significant role in this system. Both murine and human Axl has been shown to undergo proteolytic cleavage to produce a soluble isoform of the molecule [47]–[52]. Soluble Axl has been detected in the conditioned medium of tumor cells and in the serum of humans and mice under a number of pathologic conditions including cancer [47]. In addition, several reports suggest that Protein S, Tubby, Tubby-like protein 1 and Galectin -3 are also ligands for the GAS6 receptors [53]–[55]. Each of these molecules adds complexity to the system, and may be regulated independently of each other.

How is dormancy induced? What are the mechanisms that induce dormancy and how is it maintained? Are all PCa cells that enter the bone marrow capable of becoming dormant? Do DTCs proliferate slowly in the marrow, or do they enter an arrested state and not proliferate at all? Each of these questions represents a fundamental gap in our knowledge which requires a greater understanding for successful treatments of disseminated PCa in bone to be developed. The findings of this study suggest that the expression of Axl and Tyro3 are correlated with tumor growth in animal models of PCa disease. It is our hope that we may be able to use these findings as surrogate potential markers of a dormant/proliferative state, and that with these tools we can begin to address some of the gaps that exist in our knowledge of the biology of disseminated disease.
